# A Model to Develop Chatbots for Assisting the Teaching and Learning Process

**DOI:** 10.3390/s22155532

**Published:** 2022-07-25

**Authors:** Sonia Mendoza, Luis Martín Sánchez-Adame, José Fidel Urquiza-Yllescas, Beatriz A. González-Beltrán, Dominique Decouchant

**Affiliations:** 1 Computer Science Department, CINVESTAV-IPN, Mexico City 07360, Mexico; smendoza@cs.cinvestav.mx (S.M.); fuy@computacion.cs.cinvestav.mx (J.F.U.-Y.); 2 Systems Department, UAM-Azcapotzalco, Mexico City 02200, Mexico; bgonzalez@azc.uam.mx; 3 Information Technologies Department, UAM-Cuajimalpa, Mexico City 05348, Mexico; decouchant@cua.uam.mx

**Keywords:** chatbots, extra-school tool, middle school, teaching and learning process

## Abstract

Recently, in the commercial and entertainment sectors, we have seen increasing interest in incorporating chatbots into websites and apps, in order to assist customers and clients. In the academic area, chatbots are useful to provide some guidance and information about courses, admission processes and procedures, study programs, and scholarly services. However, these virtual assistants have limited mechanisms to suitably help the teaching and learning process, considering that these mechanisms should be advantageous for all the people involved. In this article, we design a model for developing a chatbot that serves as an extra-school tool to carry out academic and administrative tasks and facilitate communication between middle-school students and academic staff (e.g., teachers, social workers, psychologists, and pedagogues). Our approach is designed to help less tech-savvy people by offering them a familiar environment, using a conversational agent to ease and guide their interactions. The proposed model has been validated by implementing a multi-platform chatbot that provides both textual-based and voice-based communications and uses state-of-the-art technology. The chatbot has been tested with the help of students and teachers from a Mexican middle school, and the evaluation results show that our prototype obtained positive usability and user experience endorsements from such end-users.

## 1. Introduction

Chatbots are computer software systems that use natural language processing to assist humans in activities of various kinds [1]. These systems usually perform searches for keywords, phrases, examples, and patterns identified in their knowledge bases and translate them into queries. As a result, chatbots provide people with information concerning products, places, services, and events in online sales services and social networks [2].

These conversational agents have been implemented in multiple sectors, e.g., entertainment [3] and e-commerce [4], to carry out several tasks, e.g., providing recommendations [5], responding to FAQ [2], and providing procedure guidance [6]. By 2024, Insider Intelligence predicts that consumer retail companies will spend $142 billion on chatbots worldwide—up from just $2.8 billion in 2019 [7]. In fact, the COVID-19 pandemic has shown that this expectation is rapidly coming true. For instance, big enterprises have had to improvise basic chatbots on their websites or through WhatsApp, in order to deal with several problems arising from the unforeseen increase in online sales [8].

Thanks to recent technological advances, schools and universities around the world are progressively investing in educational software systems, which are not meant to replace teachers but supply useful tools that allow students to achieve better academic formation [9,10,11]. Now more than ever, due to the current global health crisis, students receive an essential part of their preparation via online information, e.g., homework, class topics, and exercises. Therefore, the education sector should not be ignored, since providing timely and accurate feedback is crucial for a successful school achievement [12].

In particular, the use and development of chatbots begins to attract academic institutions [13,14], since they can be valuable help for both students and teachers to obtain and provide information about procedures, school services, and courses, among others [15,16]. Nevertheless, most of these chatbots have limited mechanisms to provide adequate support to the learning and teaching process, as they must be profitable for both students and academic staff (e.g., teachers, pedagogues, psychologists, and social workers).

A major problem that software in education faces is that of digital illiteracy. As already mentioned, the pandemic not only caused certain technological tools to boom, but also exposed how many people lack the expertise to use them in whole or in part [17]. Thus, many teachers are not sufficiently trained or confident enough to use digital elements that can help improve the teaching and learning process [18,19]. This has to do not only with their age, but also with their socio-economic level, their educational level, and the culture that surrounds them [20]. Of course, Mexican teachers are no exception in this respect, as being forced to use the Internet as the main medium to teach their classes, many were severely constrained, especially those in rural areas with limited resources [21]. Thus, to the inherent problems of educational software must be added those of digital illiteracy, and so chatbots, which have been used as implementations to bridge this gap, have emerged [22].

In this article, we describe a novel model to develop chatbots that serve as extra-school tools. The proposed model defines key components to allow a resulting chatbot to act as an intermediary between students and the personnel involved in the educative process. The main functionalities provided by such a chatbot include facilitating communication, completing partial information, making reminders, advising students, monitoring events or situations, providing useful information, and directing students’ questions and doubts towards the adequate person.

Our model also defines several roles for interaction with a chatbot (e.g., teacher, student, and administrative staff); each one accomplishes particular tasks in the teaching and learning process. Thus, for example, regarding users who play the *student* role, they can receive tips for their classes, plus reminders of project deadlines and exam dates. For the *teacher* role, they can receive questions from students and recommend exercises and supplementary material to reinforce particular topics. To provide an easier means of interaction for less tech-savvy people, our model defines components with voice and text user interfaces.

To validate the proposed model, we have designed and implemented a chatbot as a multi-platform application, using state-of-the-art technologies. The resulting chatbot was tested by students and teachers from a Mexican middle school, who focused on evaluating the usability and user experience (UX) of this prototype. In general, our results show that the chatbot has obtained favorable ratings.

This article is organized in the following way. After analyzing related work (see Section 2), we explain the background of the proposal presented here (see Section 3). Then, we describe our model for the development of chatbots as support tools for the teaching and learning process in middle schools (see Section 4). Afterwards, we briefly describe the implementation of a chatbot following our model (see Section 5). Next, we present the assessments conducted, their results, and a discussion about the end-user evaluations of the chatbot (see Section 6). Finally, we conclude the work carried out and give some ideas to improve it (see Section 7).

## 2. Related Work

The related work is organized into two categories: School Service-Oriented and Student/Teacher-Oriented. These categories differ in the granularity of the information the chatbot can receive and send. The School Service-Oriented category includes chatbots focused on more general tasks, since the target users are typically general public searching for basic information. Conversely, the Student/Teacher-Oriented category encompasses more specialized chatbots that perform more precise tasks and satisfy the need for more personalized interactions.

The School Service-Oriented category includes chatbots that answer frequently asked questions (FAQs) or provide users with general information, such as educational offers, fees, procedures, processes, and schedules. Chatbots within this category are helpful for academic institutions, since they provide an automatic service to both internal and external users who require administrative and academic information. The major advantages of this kind of chatbot are constant availability, decreased workload for the staff, simultaneous attention of multiple users, and accessibility from any computer device. From this category, we can identify four features:

- **Information:** It refers to information, such as educational offers, staff contact data, and study plans, needed by people interested in becoming a student of the institution. Examples of chatbots that provide this kind of information are: CiSA [23], EASElective [24], KEMTbot [25], LiSA [26], TutorDocente [27], E-orientation [28], FIT-EBot [29], Mekni et al. [30], UMT-BOT [31], and Ranoliya et al. [2].
- **FAQ:** It concerns questions and answers that people commonly ask. This characteristic also exists in the Student/Teacher-Oriented category. Works like CiSA [23], TutorDocente [27], FIT-EBot [29], Mekni et al. [30], Ranoliya et al. [2], DINA [6], and Lee et al. [32] are examples of chatbots that include FAQs.
- **Procedures and processes:** It is a guide for students to perform administrative policies, e.g., how to enroll in a class or what requirements are needed to get certified, and even steps of the admission process. This characteristic is also present in the Student/Teacher-Oriented category. Some examples of these chatbots are: TutorDocente [27] and UMT-BOT [31].
- **Schedule:** It is related to specific information on academic activities, such as events, calls, and evaluations. The chatbot EASElective [24] can be mentioned as a representative of this characteristic.

The Student/Teacher-Oriented category includes chatbots that interact with both students and teachers. From this category, we can find seven features:

- **Evaluation:** It refers to evaluation instruments for students, e.g., homework, quizzes, exams, essays, and practices. Examples of chatbots with this feature are: KEMTbot [25], CHARLIE [33], Lecturer’s Apprentice [34], T-Bot/Q-Bot [35], NLAST [36], Tribubot [37], Bigham et al. [38], LTKA-Bot [39], QuizBot [40], and Ikastenbot [41].
- **Feedback:** The system provides students with feedback about their progress in class. As examples, we can mention Lecturer’s Apprentice [34], T-Bot/Q-Bot [35], NLAST [36], Tribubot [37], LTKA-Bot [39], QuizBot [40], Ikastenbot [41], Gómez Róspide and Puente [42], Chatbot [43], and Nguyen et al. [44].
- **Q&A:** People can ask specific questions to the chatbot, which can supply concrete context-based answers. Chatbots that exemplify this characteristic are: Lecturer’s Apprentice [34], T-Bot/Q-Bot [35], NLAST [36], Tribubot [37], LTKA-Bot [39], QuizBot [40], Gómez Róspide and Puente [42], Chatbot [43], Nguyen et al. [44], Bala et al. [45], Infobot [46], Dutta [47], Niranjan et al. [48], Reyes et al. [49], and Sreelakshmi et al. [50].
- **Reports:** The system provides teachers with details about the academic progress of their students. To illustrate this feature, we mention Ikastenbot [41].
- **Subjects:** In this case, the system can interact with the students about the classes they have registered. TutorDocente [27], Lecturer’s Apprentice [34], T-Bot/Q-Bot [35], Tribubot [37], LTKA-Bot [39], Gómez Róspide and Puente [42], Chatbot [43], and Infobot [46] are chatbots that allow conversations of this type.
- **Support:** It provides students with some type of assistance, e.g., how to use laboratory equipment. As a representative of this feature, we can name TutorDocente [27].
- **Tutorships:** It is about clarifying doubts about specific topics by providing students with some form of orientation, e.g., the complexity of binary search algorithms. Among these chatbots, we can mention TutorDocente [27], Lecturer’s Apprentice [34], T-Bot/Q-Bot [35], NLAST [36], Tribubot [37], LTKA-Bot [39], QuizBot [40], Gómez Róspide and Puente [42], Chatbot [43], Infobot [46], Doly [51], and CultureBot [52].

Most chatbots only fall into one category, but in many we can detect more than one feature in common. This fact is natural, as a complete solution must consider multiple functions to become truly valuable. From our review, only KEMTbot [25] and TutorDocente [27] are simultaneously within School Service-Oriented and Student/Teacher-Oriented categories. This trend is due to the technological development of chatbots, i.e., as new frameworks, libraries, and other implementation elements emerge, it is easier to develop chatbots rich in functionality, leaving behind agents that simply serve as “answering machines” (like the majority in the School Service-Oriented category). However, this also presents a problem, as it is possible to launch a chatbot full of features without any of them actually working well.

In this way, academic environments really represent a challenge for these types of systems, as they should be thought of as tools that help all those involved in the teaching and learning process, making their work easier and supporting them with their problems. To achieve this goal, all the features to be implemented must work together in harmony, as we intend in our proposal.

## 3. Background

In this section, we summarize the background of the proposal described in this article, which is intended to be a generic solution [53].

In our previous work, we used the Google Design Sprint technique [54] in order to reach a feasible solution quickly. This technique is a way to create valuable products, since they are not just aesthetically pleasing and usable, but also they create a way of thinking and a change of skills [55].

The main goal of our former work was to develop a software tool intended for schools. Thus, students can have extra support for their courses, a source of information about school policies, and an alternative form of interaction among end-users. To meet this objective, our university established a collaboration agreement with a Mexican middle school within the framework of a particular project in charge of these institutions.

We approached this technological development as follows. Firstly, we spent some days becoming acquainted with the most typical school processes and procedures. We also conducted interviews with main actors. As a result of those activities, we designed three personas who portray the critical roles that end-users of the software tool can play: student, teacher, and administrative staff.

To find the characteristics and requirements of each user role [56,57], we refined our interviews and then applied this knowledge to each persona in user stories format [58]. Then, using the personas and their user stories, we discussed several scenarios.

It should be noted that as part of our interviews, we learned that the school had already tried to implement a Moodle-based system during 2015. The adoption of this system was an initiative of some teachers and was used for a few months. However, this endeavor was not successful for two reasons: (1) there was no technical manager with sufficient expertise to maintain the system and add options as needed, so it became obsolete, and (2) the low participation of most of the teachers. Although they were open enough to the idea of integrating a digital tool into their classes, more than 60% described themselves as digitally illiterate, as their experience was limited to basic tasks such as surfing the internet (e.g., consult the Wikipedia) or using a social networking application on their smartphones.

Thus, we had a scenario with several constraints. The school needed a system that was low-maintenance, attractive, and simple enough for everyone involved to use and be motivated to do so. It was clear that making a traditional application entirely form-based was not a viable option, as it would require staff training and would be too costly for a public school to develop and maintain. In addition, because of the past failed implementation, we had more reluctance from teachers, which would not help the acceptance of the new tool [59]. Therefore, drawing on the requirements and characteristics of our personas, we decided that a likely way forward would be to develop a chatbot, as all stakeholders were familiar with applications such as WhatsApp. Additionally, this kind of system has already been successfully implemented in other educational contexts [60,61].

Once the chatbot solution was chosen, a rapid implementation was carried out, in order to realize to what extent this solution satisfied end-user’s requirements, an outline of how it would be to develop the chatbot entirely, and the pertinence of the selected technology. If we had opted, for example, for non-functional prototypes, perhaps the tests would not be conclusive about whether a chatbot was a suitable solution or not.

Finally, the former tests with end-users were conducted. Thus, we assessed the workload perceived by the participants after having performed some tasks. Eight individuals from the middle school were gathered: two teachers (one 36 years old man and one 28 years old woman) and six students—three females and three males between 14 and 15 years old, who had not used a chatbot. Thus, to perform our tests, we started letting them interact with the chatbot’s user interface for a few minutes, in order to become familiar with the different widgets and their functions. Afterwards, we asked them to complete specific tasks according to their roles. As shown by our results, the chatbot-based solution seems to be a good option, as anyone can use it, many academic processes can be systematized, it will always be available, and it allows end-users to participate accordingly.

Although these incipient results demonstrated that a chatbot could be a useful tool in our scenario, they also revealed that we needed to come up with a much more robust interaction design scheme, as text- or voice-only interaction can be confusing or tiresome in some respects, e.g., they are more prone to input errors [62]. We needed a hybrid tool: a conversational agent flexible enough to help end-users without being costly to develop and mechanisms that mirror everyday tasks in familiar environments.

## 4. A Model for Educational Chatbots

In this section, we describe the main components of a novel model for developing chatbots that support the teaching and learning process in Mexican middle schools.

**Figure 1:**
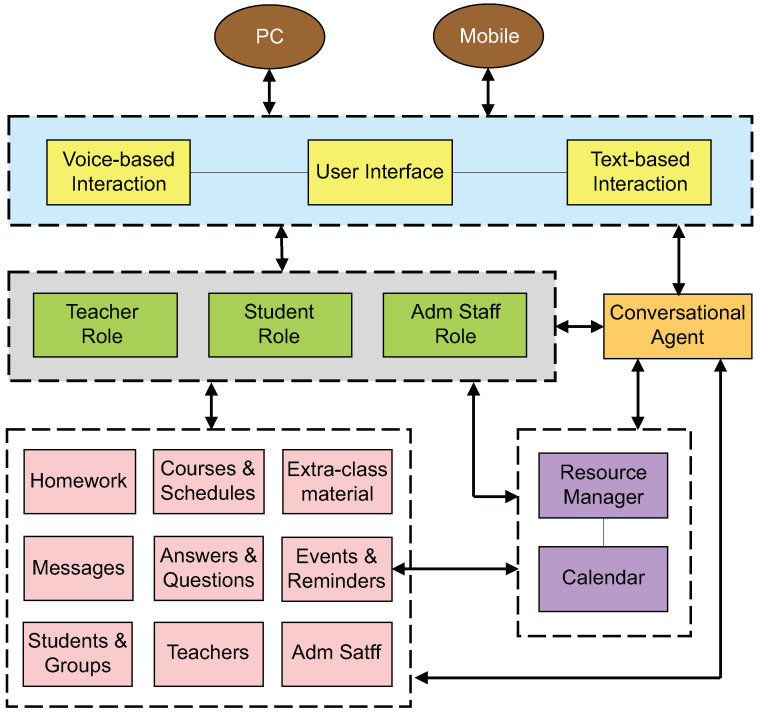
A model for a chatbot assisting the teaching and learning process in middle schools.

As shown in Figure 1, our model provides developers with a *user interface* component, which offers two possible interaction modes: *voice-based* and *text-based*. In this way, end-users can choose their preferred mode to express themselves and communicate with other end-users or with the *conversational agent*.

End-users interact with the components of the model according to a role. As mentioned before, we identified three primary roles: *student*, *teacher*, and *administrative staff*, so we defined a component that manages the specific activities of each role. In this way, by adding new roles or suppressing those that are no longer useful, our model can be easily maintained.

The *teacher role* component aims to allow teachers to organize their activities in and out of classes, to help them communicate better with their students, and to improve their classes and their work with the administrative staff. Through this component, teachers are able to visualize all their information classified by group and grade, upload extra-material for their classes, see the questions and doubts of their students, post notices and reminders for their students, and note the students’ strengths and weaknesses.

The *student role* component intends to help students communicate with their teachers, improve their learning, and share their problems and concerns with their teachers or other members of the academic staff, e.g., the psychologist, pedagogue, or social worker. Via this component, students can send their homework to their teachers; know the schedule of their classes, exams, and other important events; see extra material and presentations of the topics covered in class; send messages to their teachers; and check their class grades.

The *administrative staff role* component aims to allow the psychologist, pedagogue, or social worker to get closer to the students, providing the required attention to those who need it most. Through this component, the administrative staff can send reminders about important dates (e.g., enrolment), upload formats for scholar procedures, and answer questions and doubts of the students.

Our model also provides *students* with additional instructional materials suggested by their *teachers*. In this way, on behalf of the teacher, the conversational agent can provide students with information about complete topics of classes, answer the FAQs of crucial school processes and procedures, make automatic event reminders, guide students in each step of their administrative work, direct students with the correct office and responsible person for each organizational task, and set conversations on topics and personal matters.

The *resource manager* and *calendar* components intend to manage places, dates, and time for the programming of academic or sporting events.

The *conversational agent* component helps users carry out their activities by relying on natural language processing to ease and guide their interactions with the components of the model. Moreover, since we designed a knowledge base per functionality, developers can add, modify, or delete a knowledge base without affecting the context of others.

The remaining components are responsible for managing teacher, student, and administrative staff profiles; groups; courses; schedules; and important information exchanged among end-users, e.g., questions, answers, events, reminders, extra-class material, messages, and homework.

In the next subsections, we detail these critical components of our model.

### 4.1. Modeling User Roles

According to the Mexican middle school educational system (which also applies to other systems, such as the French one), a teacher can teach one course at least, e.g., biology, mathematics, or history. Although more than one teacher can teach the same course, a course is taught to a group of students by a single teacher (e.g., Ms. Toklas and Mr. Robinson teach Spanish for groups 1A and 1B respectively).

At least two students form a group, but one student is a member of only one group. A group of students has to take multiple courses during a school year. Therefore, a course is identified thanks to the course name, the group, and grade (e.g., English for group A in the first year). In some schools, students stay in the same classroom to take all of their courses, whereas in other schools, students have to move between different classrooms to take their courses. Since a classroom is a resource that cannot be shared between two groups of students, our model also considers this natural constraint. Thus, each course is assigned a single time and place (and vice versa) to cover both cases: mobile or stationary groups. It is worth mentioning that this property is common to all the components of our model to avoid sharing a resource that is intrinsically exclusive. In this way, the *calendar* and *resource manager* components that allow the user roles to schedule time and places can alert them to these types of issues.

**Figure 2:**
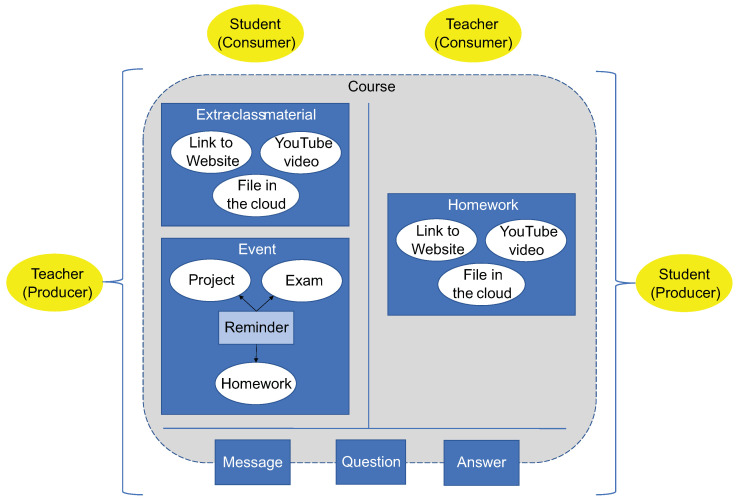
Teacher and student role models using the producer/consumer approach.

Figure 2 shows the high-level model for the *teacher* and *student* roles, which can act as both information producers and consumers. In the next subsections, we describe the user models of our proposal following the producer/consumer approach for each role identified in our former study.

#### 4.1.1. The Teacher Role

As an information producer, a teacher can:

- Create multiple extra-class materials for one or more courses. An extra-class material can even be the result of the collaborative work of several teachers. In this way, a single course can have one or more associated extra-class materials to reinforce topics covered in class. An extra-class material can be formed by links to websites, files in the cloud, and YouTube videos. To facilitate reutilization, all these elements of information can be shared among several extra-class materials.
- Generate one or more event announcements about an exam, homework, or a class project. Each event announcement has a place and time associated with it to indicate where and when the event will take place. In general, an event announcement is related to a course, which can have multiple events specified.
- Define a reminder in the context of an event announcement. In fact, an event announcement can have one or several reminders, which can be made automatically, according to a periodicity determined by the teacher (e.g., each week until 24 h before the deadline). Typically, a reminder associates with a single event. However, several event announcements can be concentrated in just one reminder, as long as the deadlines and periodicity of said events are similar or relatively close, and the recipients are the same.

As an information consumer, a teacher can:

- Accept pieces of homework for one or more courses. Typically, a piece of homework is assigned to a specific teacher who is responsible for reviewing and grading it. It is important to point out that the conversational agent takes care of informing the correct teacher that homework is ready, once the creator (i.e., a student or a group) has established that it is in the “completed” state.
- Receive statistical reports of student performance for the teacher’s courses.

#### 4.1.2. The Student Role

As information producer, a student can:

- Create multiple pieces of homework for one or more courses. A piece of homework can be the result of work done by an individual or by two or more students. A course may require the student to complete at least one piece of homework. Like extra-class material, a piece of homework can contain one or more links to websites, files in the cloud, and YouTube videos.

As information consumer, a student can:

- Accept extra-class materials for one or more courses. An extra-class material is intended for a single student, a specific group of students (e.g., topic “Usage of Passive Voice in English” for all or part of group C in the second year) or even some groups of students (e.g., groups A, B, and C in the second year). It is important to mention that the conversational agent takes care of informing the correct students that an extra-class material is ready, once the creator (i.e., a teacher or a group) has established that it is in the “completed” state.
- Receive an announcement or a periodic reminder of an academic, administrative, or athletic event. In general, an event announcement and its reminders are intended for an entire group of students, all or some groups of the same grade, specific groups of different grades, or even all the student community of the institution.

#### 4.1.3. The Administrative Staff Role

Within this role, several subroles can be derived, since the pedagogical team is not only constituted by teachers. Other actors have critical roles in the successful academic performance of students. Thus, we identify the following subroles: *social worker*, *psychologist*, *pedagogue*, and *system administrator*.

As an information producer, an administrative staff member can:

- Manage (register, update, and delete) user profiles depending on his/her role (*system administrator* subrole).
- Manage groups and courses (*system administrator* subrole).
- Generate one or more event announcements about conferences, sports, inscriptions, etc. Unlike event announcements created by teachers that are anchored to a course, these events have broader coverage, since they are intended for several groups or even for all the students of the school. These are events that take place in the auditorium, in the gym, or in the school yard, so they are also associated with respective places, dates, and times. As mentioned above, the *resource manager* and *calendar* components of our model are responsible for ensuring that the selected places, dates, and times do not conflict with others of previously defined events (all subroles).
- Define a reminder in the context of an event announcement. A reminder created by an administrative staff member has the same characteristics of a reminder created by a teacher (all subroles).

As information consumer, an administrative staff member can:

- Receive alerts from the conversational agent about a student with emotional problems; e.g., as a result of a conversation with the student, the conversational agent can direct them to the psychologist or social worker, with prior consent of the student (*psychologist* and *social worker* subroles).
- Receive alerts from the conversational agent about a student with school achievement problems; e.g., as a result of a conversation with the student and the analysis of their grades, the conversational agent can inform the pedagogue or social worker about it (*pedagogue* and *social worker* subroles).
- Receive statistical reports of student performance per student or group of one or all courses (*pedagogue* and *social worker* subroles).

#### 4.1.4. Common to All Roles

As information producer, a user with any role can:

- Send voice or text messages to an individual or a group within any of the supported roles. A message may or may not be in the context of a course, but a course may be involved in none, one, or more messages.
- Ask voice or text questions.
- Create voice or text answers to questions.

A question and an answer have the same characteristics as a message, except that an answer is always anchored to a question, but the latter may go unanswered. In addition, a question may have several answers. It is important to note that when a question has not been answered for a while, the conversational agent asks the corresponding person to answer it, discard it, or even denounce it.

As information consumer, a user with any role can:

- Receive one or more message.
- Accept questions and answers.

### 4.2. Modeling Conversational Agent Functionality

**Figure 3:**
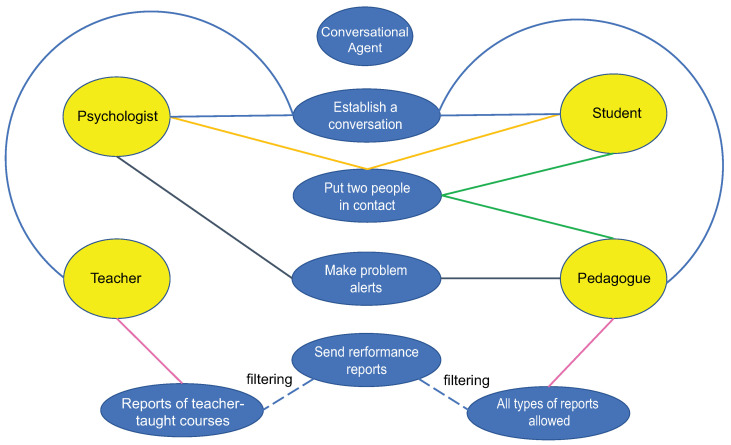
Some actions at the initiative of the conversational agent to help user roles.

As a proactive component, the conversational agent can take the initiative to perform certain actions in order to solve a problem it has detected. For instance, as shown in Figure 3, the conversational agent can determine that a student has poor performance in one or more courses from the analysis of their exam scores. Thus, it can start a dialogue with that student when detecting that he/she is active, in order to obtain more useful information (e.g., some causes of their poor performance) and encourage the student.

On the other hand, the conversational agent can also talk to a professor about some comprehension problems on a particular topic that one or more students have explicitly raised or that it has deduced from exam scores. Moreover, if these academic problems persist for a period of time, the conversational agent can put the student in contact with the pedagogue (or social worker) or only alert the latter about these issues, so that the pedagogue can take actions that can help the teacher and their students achieve a better teaching and learning process.

In a similar way, when a student has poor performance, the conversational agent can also try to talk with him/her to see whether he/she is emotionally well or not, in case the student has not taken before the initiative to talk to it about this matter. If as a result of said talk the conversational agent manages to infer (by identifying keywords in the dialogue) that the student is a victim of some type of violence at home or at school, it can put the student in contact with the psychologist (or social worker) or only alert the latter about these problems, so that the psychologist can intervene to help the student.

Furthermore, the conversational agent can take the initiative to provide a teacher with student performance statistics when the it notices that the teacher has not requested them for a while. It is important to mention that these statistical reports are automatically created. The conversational agent can also send these performance reports to the pedagogue (or social worker) so that he/she is informed of the progress of each group of students in the different courses and act accordingly.

**Figure 4:**
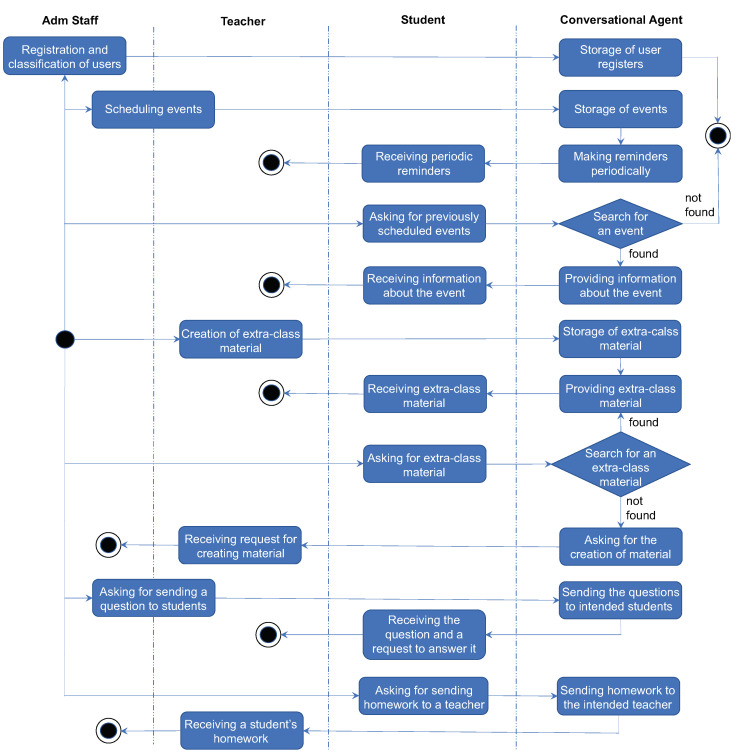
Some activities performed by user roles with the help of the conversational agent.

The conversational agent can also help complete actions performed on the initiative of user roles (see Figure 4). As we already mentioned in Section 3, our model is conceived as a hybrid proposal; i.e., the conversational agent is the guide that helps users to perform their tasks not only with text and voice but also through widgets. The interaction design we propose is intended to help people with little or no experience with any software tool, as our premise is to save them from navigating through menus and settings that can be overwhelming. Instead, we move them to a familiar environment (a chat like WhatsApp), making them perceive it as a safer environment, hence improving the overall UX [63]. Additionally, the chat format keeps interactions concise, as the conversational agent will always make end-users provide all the necessary data no matter how they choose to initiate a task.

Figure 4 shows examples of activities that can be fulfilled by the different user roles with the help of the conversational agent. The processes of registration and classification of user profiles according to their role are carried out by the system administrator, who is a member of the administrative staff.

As mentioned before, there are some activities that can be managed by more than one role. For instance, activities such as *scheduling events* and *asking for sending a question to students* can be carried out by a teacher or an administrative staff member. For this reason, these activities encompass both roles in Figure 4.

**Figure 5:**
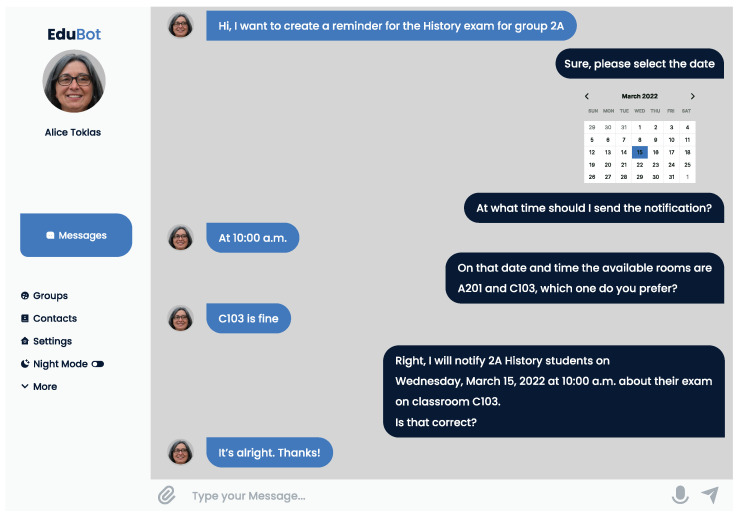
The completion of tasks is facilitated by widgets and asking for missing information; e.g., to schedule an exam, a calendar widget is used, and data that the teacher were not initially provided are required.

Both members of the administrative staff and teachers can program events, specifying date, time, and place, which are automatically stored. When a person is a teacher or administrative staff and needs to schedule an event, the conversational agent helps them with doing this task, as depicted in Figure 5. The description of the event is obviously the responsibility of the human, but the conversational agent provides a calendar showing available dates and time to make it easy to select when the event will take place. Then, according to the chosen date and time, the conversational agent checks the availability of places in the school and provides the end-user with only the available places.

Using this information, the conversational agent can provide the intended students (e.g., a specific group) with periodical reminders, freeing teachers from this cumbersome activity. In addition, at any moment, students are able to ask about a reminder for previously scheduled events. When conversational agent does not find information about the requested event, it asks the student for more information, in order to answer their request (although the conversational agent ends in Figure 4 for simplification).

Teachers can ask the conversational agent to memorize links to files, websites, and YouTube videos, and associate them with a topic. In this way, the conversational agent can provide students with extra-class materials created or approved by their teachers, and students can also ask for them. In case a student needs extra-class material about a topic, but the conversational agent did not find any information, it notifies the teacher that said student requested material.

The conversational agent allows teachers and administrative staff to send a question to a student or to all students belonging to the same group (this activity is also acceptable for students, but it is not depicted in Figure 4 for space reasons). When the conversational agent delivers a question to the intended students, it asks them for an answer. If the student ignores the response request, the conversational agent makes a certain number of periodic reminders. If these reminders fail to obtain an answer from the student, the conversational agent informs the corresponding teacher or administrative staff member about it.

Similarly, students can ask the conversational agent to send their homework to their teachers. If desired, teachers can be notified about homework delivery through alternative means, such as email. The conversational agent also sends the students acknowledgment of the good reception of their homework by their teacher. As the conversational agent receives homework from a student, the conversational agent classifies it as either “on time” or “out of time” delivery to facilitate the work of teachers.

## 5. Implementation of Our Model for Educational Chatbots

**Figure 6:**
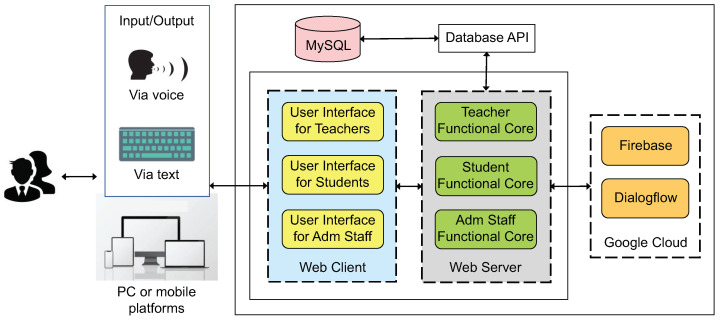
Tools and technologies used to implement the main components of our chatbot.

Figure 6 shows the tools and technologies that make up a prototype of our model for chatbots intended to assist the teaching and learning process. The developed chatbot follows a Web-based client/server approach, since it allows end-users to access the resulting chatbot anytime and anywhere using a PC or mobile platforms.

End-users can interact with the chatbot through a keyboard, or via voice. This feature is thanks to the user interface that was implemented for the three possible user roles, i.e., teachers, students, and administrative staff. The components for these roles, on both Web client-side (i.e., the user interface) and server-side (i.e., the functional core), were developed with AngularJS.

The work of processing and understanding natural language is carried out with Google cloud technologies: Firebase and Dialogflow. In particular, Firebase is in charge of storing information closely related to the chatbot, such as questions and answers. On the other hand, Dialogflow is responsible for training the model that nourishes the chatbot, which gives “life” and “behaviour” and allows communication between the chatbot and the different user roles. We also use a MySQL database to store chatbot-independent information, e.g., user data, reminders, extra-class material, messages, homework, and courses.

We created a database API which serves as an object relational map (ORM) to map the relational database to objects and facilitate their use in the components on the server side. These latter connects with Dialogflow for the conversational agent, and with Firebase for the NoSQL databases of both plain text (real-time) and complex files (cloud storage). The link between Dialogflow and Firebase occurs because Firebase provides data to the Dialogflow-hosted agent, and the agent itself stores data in Firebase.

**Figure 7:**
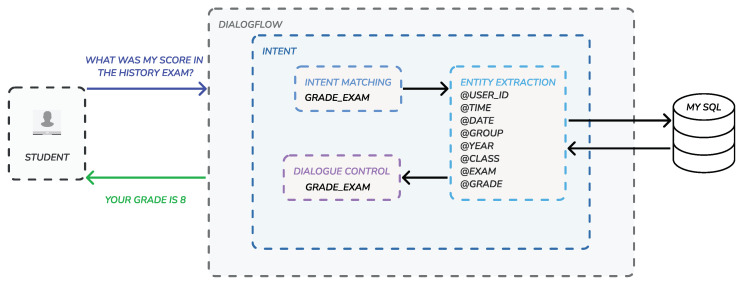
Intent is the main unit of work in Dialogflow. Thanks to their processing, it is possible to obtain entities to perform operations and hold conversations with end-users.

The connection between Dialogflow and the MySQL database is provided by intents, which are the main processes by which Dialogflow does natural language processing. When a end-user asks a question, it is processed through the three steps of an intent: (1) intent matching, which is about recognising what the end-user wants to do; (2) entity extraction, which extracts the relevant data (entities) about what the end-user wants; and (3) dialogue control, which shapes the conversation. In this way, once the intent has extracted the entities needed for an operation, these are used to perform the corresponding queries in the MySQL database, which in turn, returns the result to the intent in order to give a response to the end-user (see Figure 7).

Finally, to improve the reproducibility of our project, we published this implementation in a GitHub repository: https://github.com/jfuy/CinvestavCHATBOT (accessed on 21 June 2022).

## 6. Evaluation of Our Chatbot with End-Users

In this section, we describe the assessment that was carried out to evaluate our prototype. First, we explain the context of the trials. Next, we report the results of the tests (see Section 6.1). Finally, we analyze the significance of the obtained data (see Section 6.2).

To evaluate the proposed model, we put our chatbot prototype to the test. With the help of teachers, students, and the User Experience Questionnaire (UEQ) [64], we evaluated the UX of our prototype. We chose to do tests of this kind because we were interested in the reception of the end-users to this type of technology, the way we implemented the functionalities, and the general attitude towards the components of our model.

We chose UEQ because it is a questionnaire that people can answer quickly and considers hedonic and pragmatic aspects. Through a 26 series of semantic differentials (e.g., good-bad), it measures six scales [65]:

- **Attractiveness:** General opinion of the artifact. Do users like or dislike it?
- **Perspicuity:** Is it easy to get comfortable with the artifact and to learn how to use it?
- **Efficiency:** Can users solve their tasks without unnecessary effort? Does it react fast?
- **Dependability:** Does the user feel in control of the interaction? Is it reliable and foreseeable?
- **Stimulation:** Is it appealing and encouraging to use the artifact? Is it fun to use?
- **Novelty:** Is the design of the artifact imaginative? Does it catch the interest of users?

We used an opportunistic sample to recruit our participants, all from the same middle school. The group consisted of 10 teachers (7 female and 3 male) with an average age of 41.2 years and 10 students (6 male and 4 female) with an average age of 14.1 years. The teachers teach a variety of classes (e.g., Computer Science, Mathematics, History, Geography, and Spanish), and the pupils belonged to the 2nd and 3rd years. In all cases, they had no experience using educational chatbots, only brief encounters with casual chatbots such as those used in customer service.

To minimize non-relevant stimuli, we conducted our tests in a quiet environment so that participants felt comfortable. Each session was conducted at around 11 a.m. for 20 days (one participant per day). Each volunteer participated individually in the assessment accompanied by an on-site moderator.

To do this, once we gave them a little time to familiarize themselves with the chatbot (on a PC), we asked the participants to carry out the following tasks:

- Ask something related to a class (e.g., when is the next History test?).
- Ask the chatbot for help on a particular topic (e.g., can you help me to solve equations?).
- Send a file to another user (e.g., I want to send my Geography assignment).

After completing the three tasks, the participants answered the questionnaire; each assessment took around 45 min in total. Thus, both teachers and students had a sample of the functionalities implemented in our prototype.

### 6.1. Results

The UEQ employs a 7-point scale ranging in score from −3 (horribly bad) to 3 (extremely good) to gather participants’ ratings for each semantic differential; i.e., if a participant selects number 1 on the scale of a differential, this will be given score −3; on the other hand, if the participant answers 7, the score will be 3 [64].

**Figure 8:**
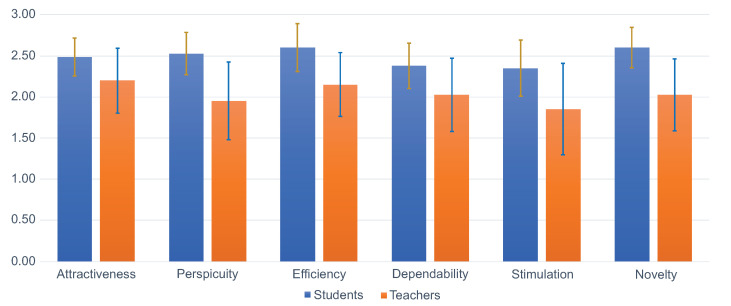
Scales results.

Table 1 and Table 2 represent the means and standard deviations for each scale of the results obtained from the groups of students and teachers. In the same way, they also contain the confidence intervals of the results. Figure 8 is the graphic representation of these results.

The confidence interval is a measure for the estimation precision of the scale mean. The smaller the confidence interval is, the higher the estimation precision and the more we can trust the results. The width of the confidence interval depends on the number of available data and on how consistently the persons judged the evaluated product. The more consistent their opinion is, the smaller is the confidence interval [64].

Reliability was evaluated by assessing the internal consistency of the UEQ scales. The Cronbach’s alpha coefficients of the instrument were classified, as shown in Table 3, for students and teachers, respectively.

The scales of the UEQ can be grouped into pragmatic quality (perspicuity, efficiency, dependability) and hedonic quality (stimulation, originality). Pragmatic quality describes the task-related quality aspects, whereas hedonic quality explains the non task-related quality aspects [66]. Table 4 shows the means for both qualities.

The UEQ also provides a benchmarking tool, which contains the data of 21,175 persons from 468 studies concerning different products (e.g., business software, webpages, webshops, and social networks). The benchmark classifies a product into five categories [66]:

- **Excellent:** in the range of the 10% best results.
- **Good:** 10% of the results in the benchmark data set are better, and 75% of the results are worse.
- **Above average:** 25% of the results in the benchmark are better than the result for the evaluated product; 50% of the results are worse.
- **Below average:** 50% of the results in the benchmark are better than the result for the evaluated product; 25% of the results are worse.
- **Bad:** in the range of the 25% worst results.

**Figure 9:**
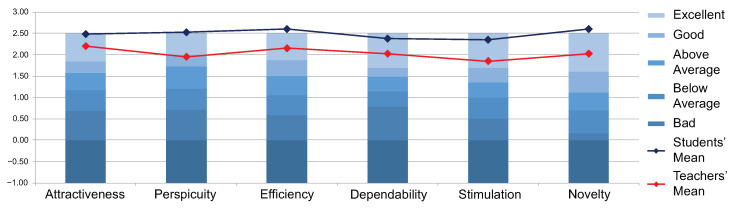
Benchmark results.

Our results on the benchmark are found in Figure 9, for students and teachers.

### 6.2. Discussion

Certainly, our model for chatbots aims to improve the teaching and learning process by automating and digitizing everyday processes. In that respect, it is no different from any other software engineering case. However, our context has an element that cannot be ignored, i.e., technological illiteracy on the part of many teachers and administrative staff. Thus, our user-centered design approach required end-user evaluations, since an UX assessment allows us to learn about user perceptions: the usability that indicates how easy it was to use our chatbot and the hedonic aspects that indicate the emotions provoked by the use of our application. These tests showed whether the design of our model is coherent with the problems encountered and whether the implementation was adequate or not.

We aimed to create a truly useful tool that can be used on a daily basis in the classroom and grow according to the needs of the community as they arise. This is a big challenge, as we not only rely on elements of natural language processing and understanding, but we had to develop mechanisms that make the tasks in the teaching and learning process easier. This is why the UX evaluation was vital, as it allowed us to know how end-users appreciated our chatbot; the last thing we wanted was to end up with was a cumbersome application that no one would like to use.

For UEQ scales, averages are usually between −2 and 2; i.e., participants do not tend to rate with extremes [66]. Taking this into account, our results were positive for both groups. As can be seen in Table 1 and Table 2, all averages were greater than or close to 2. In the case of the students, we had better ratings, since the confidence intervals are narrower than those obtained with the teachers. We attribute this to the fact that students have much more experience with software applications of various kinds, so they are more comfortable experimenting with a new system. Qualitatively, we can say that this is consistent with our general observations of the participants: the teachers were much more cautious when performing the test tasks. Nevertheless, both groups yielded consistent data, as all Cronbach’s alpha coefficients were greater than 0.7 (see Table 3).

Our positive results are reflected jointly in the pragmatic and hedonic qualities (see Table 4). This indicates that the implemented mechanisms were well received as task solvers and as items that are enjoyable to use.

The last aspect that UEQ provides is the benchmark (see Figure 9). We can highlight that the students’ averages appeared in the “Excellent” category in all cases. As for the teachers, it was the same; only the Perspicuity scale was in the “Good” range. Of course, these results are not defined enough, as the context in which all the studies were made is missing. We can only mention the interpretation offered by UEQ; i.e., our scales were in the top 10% range.

Another reason to chose an end-user evaluation was because of the nature of our context. As can be seen in Section 2, educational chatbots can offer a variety of features, so they can focus on a specific task with limited functionalities, or they can have more mechanisms without any particular specialty. Of course, we cannot call any of these approaches inherently good or bad, but it will depend on the needs of the community the chatbot is targeting.

What we did observe in our state-of-the-art research is that there is a certain epistemic immaturity in the field of educational chatbots. Most of the research does not detail the models they developed, so the mechanisms cannot be easily replicated. Additionally, there is the kind of assessments that were applied: in the same subject, there are tests and results that focus on educational performance, others that prefer the approach of software quality, and still others that analyze the usability and UX of their systems (as in our case). If the difference on granularity of the descriptions of the developments is added to this, the result is that presenting a work of this nature is complicated.

In our case, we decided to detail the characteristics of our model, not only because of the technical effort involved, but also because it is the best way we found to represent the specific needs of our study community, i.e., how an everyday task can be solved through a chatbot.

## 7. Conclusions and Future Work

In this article, we proposed a novel model for the development of chatbots that assist in the teaching and learning process in Mexican middle schools. This proposal defines three user roles which have well-defined activities that guide the user–chatbot interaction. We devised the roles in the first phase of our project, through “Google Design Sprint,” which allowed us to obtain the requirements and wishes of end-users.

Our commitment was to create a highly flexible model with components that allow close interoperability, but at the same time, are sufficiently defined and independent for organic evolution to be possible. Another of our objectives was to create a modular design for the roles, since the interaction with the chatbot ultimately depends on the role that the end-user takes.

We consider this an interdisciplinary work, which is why it was not easy to choose the type of tests to be carried out. We think that the most holistic scenario would be to implement and test with end-users. This would allow us not only to have first-hand experience of the software performance, but also whether it adequately met the requirements of end-users.

Regarding the results of the UEQ test, it can be said that both teachers and students found the chatbot a practical and friendly tool, considering that it is the first time they used such technology. The results in the efficiency and dependability dimensions were very positive. These two dimensions were the most important, since they indicate whether the chatbot was the correct tool for the scenario in question (Mexican middle schools), as well as whether the implementation covered the requirements stated. We had a small sample, but the obtained results were reliable and auspicious. Additionally, thanks to the Google technologies used in our implementation, a low-maintenance system was achieved, as Dialogflow agents are self-training [67].

A chatbot developed with our model can be a useful tool for the teaching and learning process. A big problem that many teachers face (at least in our experience) is that they are to some extent digital illiterate, so using natural language as a means of interaction may be a more straightforward process. We plan to continue working closely with members of the middle school to improve our model for educational chatbots. Similarly, our purpose is to integrate the chatbot functions into an application more familiar to end-users, such as WhatsApp or Facebook Messenger.

In addition, since our model involves handling potentially sensitive data (e.g., mental health issues), it is important to establish an information flow policy. End-users must be assured that their information will not be leaked by third parties due to bugs or misconfigurations [68]. This will ensure that particular materials are only accessible to the right people; e.g., reports on violence or depression should only reach the hands of psychologists.

## Figures and Tables

**Table 1:** Students results (n=10). Confidence interval (p=0.05) per scale.

Scale	Mean	Std. Dev.	Confidence	Confidence Interval	
Attractiveness	2.483	0.372	0.231	2.253	2.714
Perspicuity	2.525	0.416	0.258	2.267	2.783
Efficiency	2.600	0.474	0.294	2.306	2.894
Dependability	2.375	0.445	0.276	2.099	2.651
Stimulation	2.350	0.555	0.344	2.006	2.694
Novelty	2.600	0.394	0.244	2.356	2.844

**Table 2:** Teachers results (n=10). Confidence interval (p=0.05) per scale.

Scale	Mean	Std. Dev.	Confidence	Confidence Interval	
Attractiveness	2.200	0.637	0.395	1.805	2.595
Perspicuity	1.950	0.762	0.472	1.478	2.422
Efficiency	2.150	0.626	0.388	1.762	2.538
Dependability	2.025	0.721	0.447	1.578	2.472
Stimulation	1.850	0.899	0.557	1.293	2.407
Novelty	2.025	0.702	0.435	1.590	2.460

**Table 3:** Cronbach’s alpha coefficients.

Scale	Students	Teachers
Attractiveness	0.81	0.92
Perspicuity	0.80	0.92
Efficiency	0.94	0.88
Dependability	0.89	0.94
Stimulation	0.84	0.96
Novelty	0.76	0.92

**Table 4:** Pragmatic and hedonic qualities.

Scale	Students	Teachers
Pragmatic Quality	2.50	2.04
Hedonic Quality	2.48	1.94

## Data Availability

The data presented in this study are available on request from the corresponding author.
